# Polymorphic ventricular tachycardia and cardiac arrest from abiraterone-induced hypokalemia: a case report

**DOI:** 10.1186/s13256-024-04513-3

**Published:** 2024-04-16

**Authors:** Jessica Mao, Allison Komatsu Chang, Stephen Chin, Komal Preet, Nare Torosyan, Sarmen Sarkissian, Joseph Ebinger

**Affiliations:** 1 Department of Cardiology, Smidt Heart Institute, Cedars-Sinai Medical Center, Los Angeles, CA USA; 2 Department of Pharmacy, Cedars-Sinai Medical Center, Los Angeles, CA USA; 3 Department of Internal Medicine, Cedars-Sinai Medical Center, Los Angeles, CA USA; 4 David Geffen School of Medicine at the University of California, Los Angeles, Los Angeles, CA USA; 5 Department of Hematology-Oncology, Memorial Care, Long Beach, CA USA

**Keywords:** Arrhythmia, Medication side effect, Electrolyte derangement, Critical care

## Abstract

**Background:**

Polymorphic ventricular tachycardia (PMVT) is an unstable and often fatal cardiac tachyarrhythmia. While there are many causes of this rhythm, including electrolyte imbalances, ischemia, and genetic disorders, iatrogenic etiologies are important to recognize. Abiraterone is an androgen synthesis antagonist effective in treating prostate cancer, but here we describe a case of severe hypokalemia secondary to abiraterone resulting in polymorphic ventricular tachycardia and cardiac arrest. While this is a potential adverse effect of the medication, severe hypokalemia causing polymorphic ventricular tachycardia and cardiac arrest, as seen in our patient’s case, has not been described.

**Case presentation:**

A 78-year-old African-American man with history of prostate cancer presents with polymorphic ventricular tachycardia and cardiac arrest. After resuscitation, he was found to be severely hypokalemic and refractory to large doses of repletion. Evaluation of secondary causes of hypokalemia identified the likely culprit to be adverse effects from prostate cancer treatment.

**Conclusion:**

A broad differential diagnosis for polymorphic ventricular tachycardia is essential in identifying and treating patients presenting in this rhythm. Here we present a case of iatrogenic polymorphic ventricular tachycardia secondary to oncologic treatment.

## Background

Polymorphic ventricular tachycardia (PMVT) is an unstable and often fatal arrhythmia characterized by wide and varying QRS complexes. Management with urgent defibrillation and following the Advanced Cardiac Life Support protocol is standardized, but identifying and reversing or mitigating the underlying cause is essential for prevention of further morbidity and mortality. Etiologies of PMVT can be divided into reversible and irreversible causes, include coronary ischemia, electrolyte derangements, and predisposing genetic conditions. Consideration should also be given to secondary causes. While different presentations of PMVT may present with morphologically similar electrocardiographic findings, different types respond to different treatments. In some cases, appropriate treatment for one cause can worsen another [1].

Abiraterone is an androgen synthesis antagonist that affects the adrenal steroid biosynthesis pathways. It has been shown to be effective in prolonging survival in prostate cancer. However, resultingly, given its mechanism of action, hypokalemia and other electrolyte derangements can be rare but serious adverse events [2]. Herein, we present a case of a patient presenting with PMVT secondary to severe hypokalemia from abiraterone therapy.

## Case presentation

**Fig. 1:**
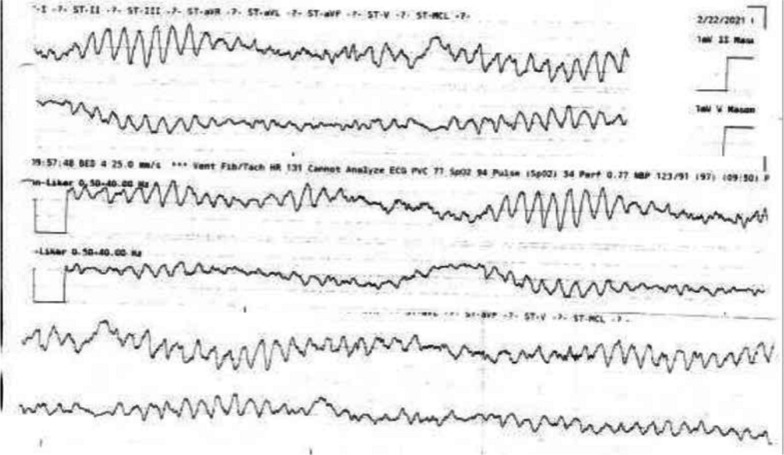
Telemetry showing polymorphic ventricular tachycardia

A 78-year-old African-American male drove himself to the emergency department with dizziness. After a syncopal event in the parking lot, he recovered spontaneously and walked into the hospital. During initial evaluation, the patient again lost consciousness and was found to be in polymorphic ventricular tachycardia (Fig. 1). Cardiopulmonary resuscitation was initiated, magnesium and amiodarone were administered, and the patient was defibrillated four times before return of spontaneous circulation (ROSC).

After ROSC, he was noted to have a heart rate of 30 beats per minute with repeated episodes of nonsustained polymorphic ventricular tachycardia. Intravenous dopamine infusion was initiated, followed by temporary transvenous pacing. The patient was then transferred to a tertiary care hospital for further management.

### Past medical history

The patient’s past medical history was significant for atrial flutter, hypertension, and prostate cancer. His atrial flutter was rate controlled without atrioventricular nodal blockade, and he was anticoagulated with apixaban for a CHA_2_DS_2_–VASc score of 3.

Prostate cancer had been diagnosed 12 years prior to his current presentation, with metastases to the cervical spine, as well as left greater trochanter, ankle, and toes. Treatments included triptorelin pamoate, diethylstilbestrol, enzalutamide, sipuleucel-T, and, most recently, abiraterone, an androgen synthesis antagonist, coadministered with prednisone. The patient had notably stopped prednisone several months prior when his prescription ran out.

Hypertension was initially poorly controlled, with hospitalization for hypertensive urgency 1 year prior. While his blood pressure had subsequently been controlled with amlodipine and losartan, he developed lower extremity edema, and amlodipine was switched to chlorthalidone approximately 9 months prior to his current presentation.

### Differential diagnosis

The differential diagnosis for a patient presenting with polymorphic ventricular tachycardia can be found in Table 1. For this patient, most pertinent diagnoses include ischemic heart disease, a particularly important consideration among individuals with both modifiable (hypertension) and nonmodifiable (advanced age and male sex) risk factors for coronary artery disease. Given the association of polymorphic ventricular tachycardia with prolonged QT intervals, consideration must also be given to acquired or inherited conditions including electrolyte derangements, specifically hypomagnesemia and hypokalemia, as well as familial long QT syndromes.

**Table 1:** Causes of polymorphic ventricular tachycardia

With prolonged QT interval	Without prolonged QT interval
Congenital syndromes	Short QT syndrome
Drug induced	Early repolarization (J wave) syndrome
Bradycardia-induced	Brugada syndrome
Post tachycardia	Coronary ischemia
Post coronary infarction	Idiopathic
Takotsubo	
Hypokalemia (and other electrolyte abnormalities)	
Hypogonadism	

### Investigations

**Fig. 2:**
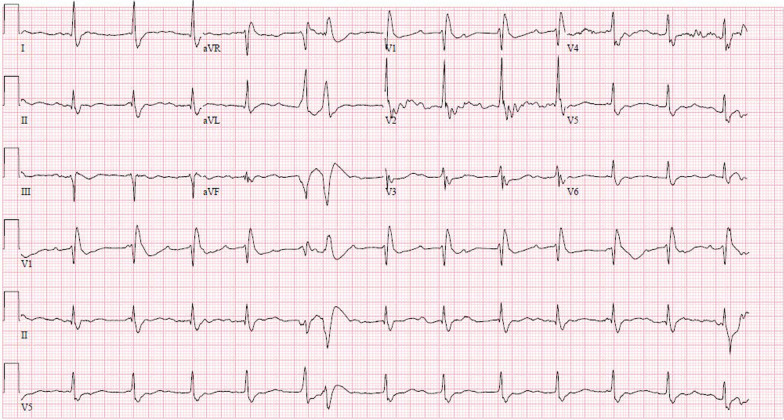
Initial electrocardiogram showing junctional rhythm with premature ventricular contractions

An ECG was performed, which demonstrated a regular accelerated junctional rhythm with premature ventricular contractions and right bundle branch block, with QT and QTc of 492 milliseconds and 568 milliseconds, respectively (Fig. 2). Initial laboratory values following ROSC are found in Table 2. A bedside transthoracic echocardiogram demonstrated a mildly depressed left ventricular ejection fraction at 48%, without wall motion abnormalities or significant valvular lesions.

**Table 2:** Laboratory evaluation results at presentation

Laboratory test	Result	Normal reference values
Arterial blood gas (on 100% FiO_2_)		
pH	7.45	7.35–7.45
pO_2_	425 mmHg	75–100 mmHg
pCO_2_	37 mmHg	35–45 mmHg
Sodium	138 mmol/L	136–145 mmol/L
Potassium	1.3 mmol/L	3.5–5.1 mmol/L
Chloride	112 mmol/L	99–107 mmol/L
Bicarbonate	14 mmol/L	21–31 mmol/L
Blood urea nitrogen	15 mg/dL	7–25 mg/dL
Creatinine	0.7 mg/dL	0.7–1.3 mg/dL
Magnesium	1.7 mg/dL	1.5–2.3 mg/dL
Troponin-I	0.07 ng/mL	< 0.04 ng/mL
B-type natriuetic peptide	132 pg/mL	< 100 pg/mL
Lactate	3.7 mmol/L	0.5–2.2 mmol/L
White blood cells	12.64 k/μL	4.0–11.0 k/μL
Hemoglobin	12.5 g/dL	13.9–16.3 g/dL
Hematocrit	37.4%	43.5–48.0%
Platelets	332 k/μL	150–450 k/μL

Despite receiving between 100 and 300 mEq of potassium repletion daily, the patient experience persistent hypokalemia (Table 3), prompting exploration of potential secondary etiologies. Renin activity was measured at 0.21 ng/mL per hour (normal range 0.25–5.82 ng/mL per hour), plasma aldosterone level was undetectable, adrenocorticotropic hormone (ACTH) level was 54 pg/mL (normal range 6–50 pg/mL), and morning cortisol level was 12.5 ug/dL. At this time, potassium was 2.7 mEq/L. A total of 24 urine potassium collection resulted inappropriately normal at 112 mmol per 24 hours (normal range 25–125 mmol per 24 hours).

**Table 3:** Potassium repletion during hospitalization

Hospital day	Potassium administered (mEq)	Serum potassium level (mmol/L)
0		1.3
1	150	2.6
2	240	3.2
3	320	3.3
4	320	3.6

### Management

As laboratory evaluations showed no other obvious etiology of hypokalemia, it was felt the presenting electrolyte abnormalities were likely secondary to abiraterone administration without glucocorticoid, causing excess mineralocorticoid activity. The patient was started on prednisone 20 mg daily, with minimal improvement in potassium requirement (160 to 120 mEq daily). He was then started on eplerenone 25 mg daily, with reduction of daily potassium requirement to 80 mEq daily.

The patient experienced persistent bradycardia with heart rates in the 50 seconds despite normalization of serum potassium. Heart rhythms observed during hospitalization included Mobitz I, ectopic atrial beats, atrial flutter and fibrillation, intermittent sinus arrest, and persistent bifascicular block with right bundle branch block and left anterior fascicular block. The decision was made to proceed with implantation of permanent pacemaker prior to discharge. While placement of an implantable cardiac defibrillator (ICD) was discussed, it ultimately was judged to be not indicated as initial VT arrest was secondary to severe hypokalemia. Specifically, the hypokalemic trigger was felt to be reversible with changes to the patient’s medical regimen, making risk of recurrent VT unlikely.

Following correction of the patient’s hypokalemia, no further episodes of polymorphic ventricular tachycardia were appreciated. QT and QTc intervals on ECG prior to discharge improved to 466 milliseconds and 477 milliseconds, respectively, while being ventricular paced. Given his lack of anginal symptoms or ECG changes concerning for ischemia, as well as another likely etiology for his initial presentation, coronary evaluation was deferred.

### Follow-up

Abiraterone was stopped, the patient was weaned off prednisone, and he was discharged on lisinopril, eplerenone, and potassium chloride 40 mEq twice daily. The potassium was decreased to 40 mEq once daily 2 weeks post discharge and stopped completely 4 weeks post discharge visit, at which time serum K was 4.9 mEq/L.

## Discussion and conclusions

Abiraterone is a selective inhibitor of CYP17, resulting in reductions in testosterone production and is approved for the treatment of metastatic, castration-resistant prostate cancer. It has been shown to prolong radiographic progression-free survival, as well as pain progression and time to chemotherapy [2].

**Fig. 3:**
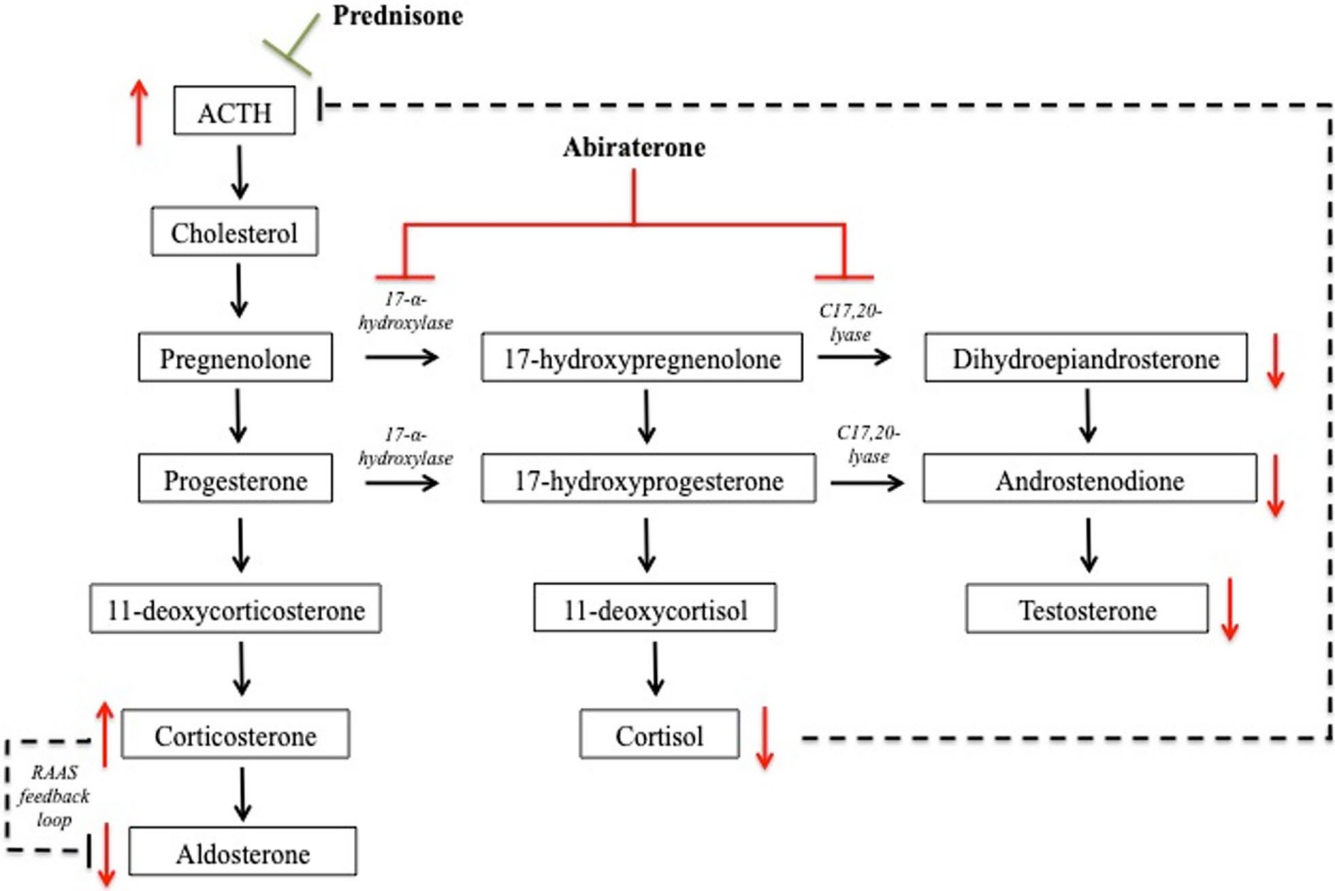
Adrenal steroid biosynthesis pathways. Abiraterone inhibits 17-α-hydroxylase and C17,20-lyase decreasing dihydroepiandrosterone and subsequent testosterone. Synthesis of cortisol, which feeds back to inhibit adrenocorticotropic hormone, is also diminished and leads to overproduction of adrenocorticotropic hormone decreased aldosterone

CYP17 carries 17-α-hydroxylase and 17,20-xylase activity. Inhibition results in blockade of the androgen biosynthetic pathway, preventing the conversion of pregnenolone to 17OH-pregnenolone and 17OH-pregnenolone to cortisol and dihydroepiandrosterone (DHEA; a precursor to testosterone) (Fig. 3). Cortisol, which feeds back to lower ACTH production, is diminished and as such, prednisone is coadministered with abiraterone to fill this role. Without glucocorticoid coadministration, abiraterone can result in an overproduction of ACTH; resultant mineralocorticoid synthesis, including corticosterone, decreases aldosterone levels via the renin–angiotensin–aldosterone feedback loop causing hypertension and hypokalemia [3].

As part of his evaluation, the patient was found to have a low renin activity level, as well as an undetectably low aldosterone level As abiraterone inhibits 17α-hydroxylase, this ultimately results in a decrease in cortisol and rise in adrenocorticotrophic hormone (ACTH). Elevated 11-deoxycorticosterone and corticosterone levels result in symptoms of elevated mineralocorticoid with inability to retain potassium in the distal collecting duct. Aldosterone levels are decreased as the result of suppression from the renin–angiotensin inhibition pathway in response to elevated corticosterone levels (Fig. 3). Hypokalemia in our patient was further exacerbated by the use chlorthalidone, initiated several months prior to presentation.

An analysis of side effects of abiraterone administration without prednisone found that nearly half of patients developed worsening controlled of hypertension, 19% required potassium supplementation, and 12% were ultimately initiated on prednisone for adverse effects of mineralocorticoid toxicity [4]. Despite being well described [5], abiraterone associated hypokalemia is typically mild, though profound hypokalemia can occur. Two cases reported in Japan , with one patient suffering severe lethargy and another seizures, were found to have serum potassium levels of 1.7 and 2.1 mEq/L, respectively [6]. Both patients were on prednisone and required increased doses of prednisone following withdrawal of abiraterone. Life-threatening cardiac arrhythmias from abiraterone-associated hypokalemia have also been described. A case report from Columbia describing nonsustained polymorphic ventricular tachycardia in a patient receiving abiraterone without prednisone and a serum potassium of 2.4 mEq/L and serum of magnesium 0.8 mg/dL [7]. Several other case reports of Torsades de Pointes have also been reported [8–12].

Given extreme potassium depletion and ongoing wasting, prednisone and eplerenone were added our patient’s regimen. Eplerenone was preferentially chosen over spironolactone, as the latter has been case reported to act as an androgen receptor agonist in androgen-depleted patients, such as those on abiraterone therapy [13]. Eplerenone coadministration with abiraterone has been shown to be noninferior to prednisone in preventing effects of mineralocorticoid excess such as hypokalemia, hypertension, and edema [14].

Beyond hypokalemia-induced arrhythmias, abiraterone is associated with atrial tachycardias, more so than other androgen deprivation therapeutics such as enzalutamide [15]. Consideration was given to abiraterone as a potential cause for his history of atrial flutter; however, review of his records demonstrated that atrial flutter first occurred prior to him receiving abiraterone.

While cardiac arrythmias are one of the most feared sequela of profound electrolyte derangements, iatrogenic causes of such changes demand attention to noncardiac medications. Here we present a case of severe hypokalemia, secondary to oncologic treatment without rescue steroid therapy, with associated polymorphic ventricular tachycardia. Routine surveillance and vigilance about medication interactions can help prevent severe consequences.

## Data Availability

Not applicable.

## Abbreviations

ACTH: Adrenocorticotropic hormone
ECG: Electrocardiogram
ROSC: Return of spontaneous circulation
